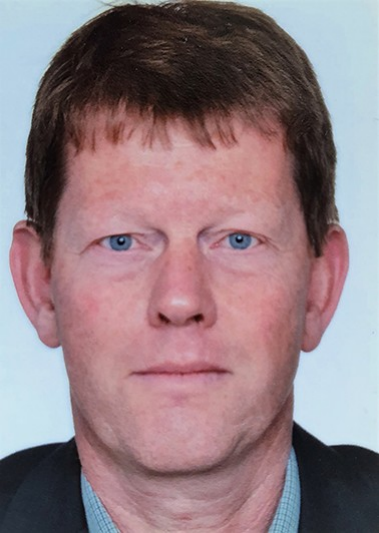# Fundamental Aspects of Ceria Supported Au Catalysts Probed by In Situ/Operando Spectroscopy and TAP Reactor Studies

**DOI:** 10.1002/cphc.202100027

**Published:** 2021-06-08

**Authors:** Ali M. Abdel‐Mageed, Shilong Chen, Corinna Fauth, Thomas Häring, Joachim Bansmann

**Affiliations:** ^1^ Institute of Surface Chemistry and Catalysis Ulm University Albert-Einstein-Allee 47 89081 Ulm Germany; ^2^ Department of Chemistry Faculty of Science Cairo University 12613 Giza Egypt; ^3^ Institute of Inorganic Chemistry Kiel University Max-Eyth-Str. 2 24118 Kiel Germany

**Keywords:** operando spectroscopy, Au/CeO_2_, XANES/EXAFS, DRIFTS, TAP reactor

## Abstract

The discovery of the activity of dispersed gold nanoparticles three decades ago paved the way for a new era in catalysis. The unusual behavior of these catalysts sparked many questions about their working mechanism. In particular, Au/CeO_2_ proved to be an efficient catalyst in several reactions such as CO oxidation, water gas shift, and CO_2_ reduction. Here, by employing findings from operando X‐ray absorption spectroscopy at the near and extended Au and Ce L_III_ energy edges, we focus on the fundamental aspects of highly active Au/CeO_2_ catalysts, mainly in the CO oxidation for understanding their complex structure‐reactivity relationship. These results were combined with findings from in situ diffuse reflectance FTIR and Raman spectroscopy, highlighting the changes of adlayer and ceria defects. For a comprehensive understanding, the spectroscopic findings will be supplemented by results of the dynamics of O_2_ activation obtained from Temporal Analysis of Products (TAP). Merging these results illuminates the complex relationship among the oxidation state, size of the Au nanoparticles, the redox properties of CeO_2_ support, and the dynamics of O_2_ activation.

## Graphical abstract

**Unravelling the mystery of Au/CeO_2_ catalysts**! Challenged by the complex nature of Au/CeO_2_ catalysts, operando X‐ray absorption spectroscopy in the near and extended Au L_III_ and Ce L_III_ energy edges is employed to decipher different aspects of the electronic and geometric features of Au NPs/clusters and CeO_2_ support. These results combined with findings from ex‐situ XPS, in situ FTIR/Raman spectroscopy and temporal analysis of products (TAP) reactor, provide a more fundamental insight into the performance of these catalysts.

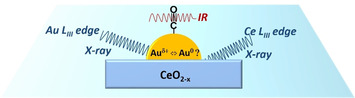

## 1 Background and Introduction

Since the discovery of their catalytic activity, Au‐based catalysts attracted enormous attention for over three decades.[^1^, ^2^, ^3^, ^4^, ^5^, ^6^] This has been motivated by the unsurpassed catalytic activity of these catalysts in a large number of oxidation and reduction reactions, already under milder reaction conditions compared to the established catalytic systems. In particular, these catalysts proved very efficient as heterogeneous catalysts for several technical processes,^[7]^ e. g., CO oxidation,[^8^, ^9^, ^10^] low temperature water‐gas shift reaction (LT‐WGS),[^11^, ^12^] aerobic alcohol oxidation,^[13]^ propylene oxidation,[^14^, ^15^] CO/CO_2_ reduction,[^16^, ^17^, ^18^] direct synthesis of hydrogen peroxide from O_2_ and H_2_,^[19]^ and acetylene hydrochlorination.[^20^, ^21^] These studies focused, on the one hand, on optimizing the catalytic performance for different catalytic applications (i. e., tailoring better Au catalysts). On the other hand, an increasing fundamental understanding of the catalytic activity and reaction mechanisms allows developing better strategies to improve the performance of these catalysts. Metal oxide supported Au nanoparticles (NPs) have been the most studied catalysts so far.^[6]^

**Figure 1:**
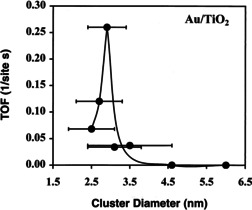
Turnover frequency (TOF) activity of Au/TiO_2_ at 300 K as a function of the average diameter of gold NPs (1.5 to 6.0 nm). Reprinted with permission from Ref. [24] © 1998 American Association for the advancement of Science (AAAS).

Interestingly, the structure‐reactivity correlation for different reactions showed a pronounced dependence of the activity on the Au particle size.[^7^, ^22^] Several studies indicated that a substantial increase of the activity of supported Au catalyst can be observed once decreasing the Au nanoparticles (NP) size well below 5 nm.^[23]^ Early work by Valden and Goodman demonstrated a volcano‐shaped dependence of the activity of Au/TiO_2_ catalysts on the average diameter of Au NPs in in a size regime <5 nm. Upon decreasing Au particle size from about 5 to 3 nm, the turnover frequency (TOF) for the CO oxidation was found to increase shapely. A further decrease of Au NP size to less than 2.7 nm, however, led to a decrease in the activity (cf. Figure 1).^[24]^

These early findings demonstrated the importance of the Au NP size on the activity of the catalysts. Besides this geometric aspect (Au particle size), several other parameters affect the catalytic activity as well, such as the method of preparation, the activation treatment, the reaction gas mixture and reaction conditions and, most importantly, the selection of the support material.^[25]^

The nature of gold species (i. e., metallic or ionic character) also has a significant impact on the catalytic performance of these catalysts. The difficulty to identify the active species was mainly attributed to the use of ex‐situ spectroscopic tools. A famous example is the nature of active gold species during WGS on Au/CeO_2_, which ranged from attributing the activity to the presence of Au^3+^ sites[^11^, ^26^] to the correlation of activity with the formation of fully reduced sub‐nanometer Au NPs.[^27^, ^28^] A possible contribution from different sites in the reaction pathway is assumed,^[29]^ but a consensus is yet not reached.

The impact of the oxide support on the performance of Au NPs is also very decisive. Early work by Behm and coworkers tried to clarify the role of oxide supports on the activity of Au NPs during CO oxidation. These studies clearly indicated an influential impact of the oxide support on the activity of resulting catalysts and concluded that metal oxide supports can be categorized as inert and active supports.^[30]^ While the first kind was recognized as non‐reducible oxides, which are specifically inert toward the interaction with oxygen under reaction conditions (i. e., oxygen activation), the second type (i. e., active oxides) was realized as reducible oxides (e. g. Fe_2_O_3_, TiO_2_, NiO_*x*_, ZrO_x_, CoO_*x*_ *)* with their ability to undergo a reversible redox reaction. The activity of reducible oxides was explained by their ability to actively adsorb, dissociate, store, and then resupply active oxygen species for completing the oxidation reaction, and Au NPs deposited on oxide surfaces lead to a further enhancement of these redox properties. Among these examples, CeO_2_ is considered as one of the most active supports for gold catalysts due to its remarkable oxidation/reduction characteristics, which makes ceria supports ideal oxygen storage materials. This property is related to their ability to release electrons from O^2−^ ions to the two adjacent Ce^4+^ atoms involving the Ce^4+^/Ce^3+^ redox couple exchange.[^31^, ^32^, ^33^, ^34^, ^35^]

Employing CeO_2_ as support for small Au NPs results in high catalytic activity for a variety of reactions including CO oxidation,[^36^, ^37^, ^38^] water‐gas shift (CO+H_2_O⇌CO_2_+H_2_),[^28^, ^39^, ^40^] and this system was even reported as highly active catalyst for the methanol synthesis from CO_2_.[^18^, ^41^] Furthermore, Au/CeO_2_ catalysts display a strong surface plasmon resonance,^[42]^ which makes them excellent candidates in photocatalytic applications.[^43^, ^44^, ^45^] Interestingly, we observed that Au/CeO_2_ catalysts behave rather similar in reactions with varying reaction gas compositions and thus under different reducing conditions (e. g., low temperature CO oxidation,[^38^, ^46^, ^47^] water gas shift,[^27^, ^28^, ^48^] and during CO_2_ reduction to methanol[^18^, ^41^]). In common, we observed that the activity increases quickly in a short activation phase followed subsequently by a continuous deactivation during reaction on‐stream. A similar behavior was also observed on Au/CeZrO_4_ catalysts during low‐temperature water‐gas shift reaction.^[29]^ The extent of deactivation differs according to the reaction, but eventually the same behavior (i. e. a continuous deactivation) can be observed. The complex structure of these catalysts and the interplay of different effects (oxidation state, particle size, support modifications) during reaction impede the physical understanding of their catalytic performance. The best approach to disentangle and understand individual roles of these effects is to employ *in‐situ*/operando spectroscopic measurements of the catalyst under reaction conditions, which gives access to the electronic and geometric characteristics of the catalyst structure simultaneously.

Recently, we studied the catalytic activity of Au/CeO_2_ during reaction with operando X‐ray absorption spectroscopy (XAS), both near the absorption edge (XANES: X‐ray Absorption Near Edge Structure) and in the extended regions (EXAFS: Extended X‐ray Absorption fine structure) of the Au and Ce L_III_ edges, together with supplementary *in‐situ* FTIR spectroscopy under identical conditions. The CO oxidation was selected as a prototype reaction for other reactions showing similar behavior on Au/CeO_2_ catalysts. Focusing on the catalyst activation/deactivation phases in correlation with changes of their structure and electronic properties is an effective approach to decipher their principle of work. The origin of deactivation of these catalysts was controversially discussed based on the following scenarios: (i) effects associated with changes of the oxidation state of Au,^[38]^ (ii) the buildup of surface poisoning carbon containing species at the perimeter of the Au‐support interface,[^49^, ^50^, ^51^, ^52^] (iii) agglomeration/sintering of Au NPs during reaction,[^35^, ^51^, ^53^] surface over‐reduction,[^41^, ^54^] and/or (iv) consumption OH groups, which presumably participate in the dominant reaction pathway.[^55^, ^56^]

The findings from the Behm group and other groups, with a focus on *in‐situ* and operando spectroscopy, will be the topic this article. We will focus both on the electronic effects/oxidation state of the metal (Au) and the CeO_2_ support and their geometric properties during the catalytic CO oxidation. The layout will include first a short description of the preparation methods of the Au/CeO_2_ catalysts (section 2). After that, we will discuss the electronic properties of Au and Ce during CO oxidation (section 3) followed by particle size effects (section 4). Then we will discuss in detail the spectroscopic evidence on the adlayer and its correlation to the formation and the role of Ce^3+^ sites (O‐vacancies) during CO oxidation (section 5). Finally, we will focus on the dynamics of formation/removal of O‐vacancy defects during reaction using TAP reactor studies (section 6).

## 2 Recipe for the Preparation of Au/CeO_2_

Different preparation methods of supported Au catalysts have been reported such as co‐precipitation, deposition precipitation, wetness impregnation, and colloidal deposition.[^8^, ^57^, ^58^, ^59^, ^60^] One of the typical challenges in preparing supported Au catalysts is, however, their high sensitivity to the method of preparation and the synthesis parameters used in each method (e. g., gold precursor, pH of the synthesis solution, ionic strength, or temperature). In other words, these parameters and different preparation methods would lead to different catalysts unless each is well controlled. This aspect may be problematic in systematic studies, where the comparison of reactivity and the catalyst structure is the ultimate goal.^[60]^

The deposition precipitation of ionic gold on metal oxide supports has been reported as one of the most controllable and also reproducible methods for preparing Au NPs in the small nanometer size range (≤5 nm), which is the desired range needed to reach the highest activity, particularly for CO oxidation,^[24]^ or WGS reaction.^[28]^ Here, auric acid (HAuCl_4_) is hydrolyzed in an aqueous solution under a controlled pH (8–9) of the desired oxide support. The strict control of pH is essential to control the Au loading and the dispersion/particle size of Au NPs.^[59]^ This process is typically achieved by addition of NaOH or Na_2_CO_3_ solution to the synthesis feed, where the selection of the base used would also affect the properties of resulting catalysts.

It should be highlighted that Au/CeO_2_ catalysts used in these studies were prepared by a deposition–precipitation (DP) method employing a high surface area CeO_2_ oxide support (HSA 15, Rhodia, 188 m^2^ g^−1^) calcined in air at 400 °C for 4 h. The CeO_2_ support was dispersed in water at a reaction temperature of 60 °C, where the gold precursor (HAuCl_4_.3H_2_O, 99.5 %, Merck) was added dropwisely at pH of 8–9, which is adjusted continuously during the loading of Au (i. e., the hydrolysis of the gold precursor) by adding a solution of Na_2_CO_3_. In the next step, the as prepared Au/CeO_2_ catalyst precipitate was washed with deionized water and subsequently dried at room temperature overnight (≥12 h). Different loadings of Au in the range between 2 and 5 wt.% were used.[^28^, ^36^, ^38^, ^46^, ^47^, ^54^] It should be mentioned that the proper selection of the surface morphology of CeO_2_ is also an additional parameter that can be controlled in the catalyst synthesis to steer the catalytic properties of resulting Au/CeO_2_ catalysts.

## 3 Electronic and Chemical Properties of Au/CeO_2_ Catalysts

X‐ray photoelectron spectroscopy (XPS) is a standard surface science technique to investigate catalysts regarding their chemical composition and their oxidation state. In most cases, however, the samples are transferred after processing to the XPS under ambient conditions (i. e., ex‐situ) which may affect the oxidation state of the catalysts due to exposure to air. While an *in‐situ* handling (without exposure to air) reduces possible modifications of the sample and (near) ambient pressure XPS (APXPS) allows measurements in gases up to some ten mbar, however,^[61]^ there is still a significant pressure gap up to ambient conditions. Up to date no APXPS experiments have been performed using Au/CeO_2_ catalysts.^[61]^ In this chapter, we will thus first present ex‐situ XPS measurements from Au/CeO_2_ catalysts (after different pretreatments and CO oxidation). In the second part, we will focus on operando X‐ray absorption measurements (XAS) at the L_III_ edges of both materials (Au and Ce), which offers insight into the chemical state of a working catalyst. This technique does not rely (like XPS and other surface sensitive techniques) on probing electrons which have an extremely small mean free path length in the mbar regime. XAS, based on tuneable X‐ray radiation (typically between 4 and 30 keV), is an element‐specific method by performing experiments (in transmission or fluorescence) at the absorption edges of the respective materials. Due to the much lower absorbance of X‐rays in gases and in solids, it allows measurements on powder catalysts under operando conditions, but, unfortunately, loses the surface sensitivity. Considering, on the other hand, the very small sizes of metal NPs, XAS (a bulk‐sensitive method) becomes quite surface‐sensitive due to the large contribution from the surface.

### 3.1 Ex‐situ XPS of Au/CeO_2_ Catalysts

**Figure 2:**
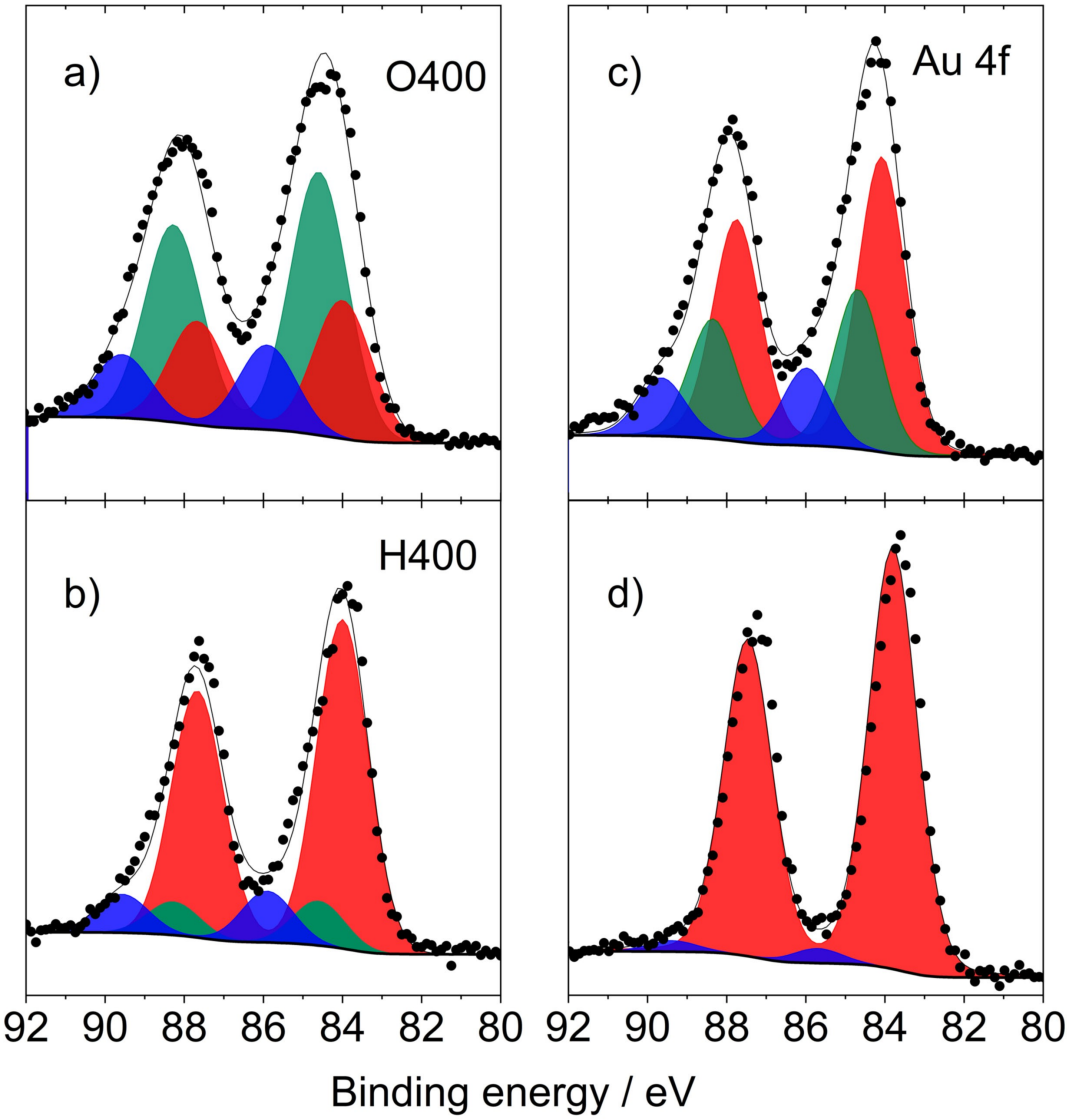
XP spectra (Au 4 f levels) of Au/CeO_2_. Left: after O400 (a,c) and H400 (b,d) pretreatments; (right) after CO oxidation. Peaks in red, green, blue: Au^0^, Au^1+^, Au^3+^ species. Reprinted with permission from Ref. [38] © 2016, Elsevier Inc.

Figure 2 displays Au 4 f spectra recorded from Au/CeO_2_ catalysts after oxidative and reductive pretreatments (left) and subsequent CO oxidation (right). Color‐coded areas illuminate the metallic (red: Au^0^) and oxidic Au species (green: Au^1+^, blue: Au^3+^). After an oxidative pretreatment (O400), the amount of oxidic Au species is much higher compared to a reductive (H400) pretreatment. Here, the abbreviations denote the composition/temperature of the gas mixture during pretreatments (e. g., H400: 10 % H_2_ in N_2_ at 1 bar and 400 °C). After CO oxidation, the amount of oxidic species has nearly vanished in case of a reductive treatment (Figure 2d) whereas still a certain fraction of oxidic species is visible in case of an oxidative pretreatment. (Figure 2c).

**Figure 3:**
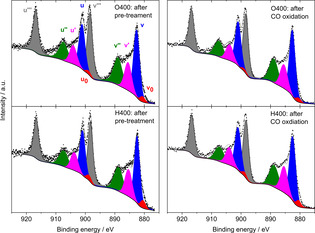
XP spectra (Ce 3d region) of Au/CeO_2_ catalysts after different pretreatments (left) and after CO oxidation. Peaks in red, pink: Ce^3+^; blue, green, grey: Ce^4+^ species. Reprinted with permission from Ref. [38] © 2016, Elsevier Inc.

The situation is different when investigating the Ce 3d core levels of the metal oxide support. The Ce 3d XPS data (Figure 3) recorded analogously to Figure 2 display nearly identical spectra before and after CO oxidation, and results are independent of an oxidative/reductive pretreatment. A deconvolution of the complex spectra shows little variation in the ratio of Ce^3+^ (red and pink areas) compared to Ce^4+^ species (blue, green, and grey).

For the assignment, we used the same model and notation as previously reported publications of the Behm group.[^38^, ^62^] This result was unexpected, we expected at least a higher amount of Ce^3+^ species after reductive (H400) pretreatments similar to the higher amount of metallic Au (cf. Figure 2). Different from the rather inert Au NPs in the Au/CeO_2_ catalysts, almost any oxygen vacancies induced in the ceria support during pretreatment/reaction most likely reacted with oxygen from the air when being exposing to ambient condition during the transport of the sample. Thus, all the XPS measurements in the Ce 3d region (Figure 3) merely display the natural amount of O‐defects in ceria and not the situation after the respective process. In the following section, we will highlight the presence of Ce^3+^ species in the ceria support by employing operando XAS measurements at the Ce L_III_ edge.

### 3.2 Operando X‐ray Absorption Spectroscopy at Catalysts

Operando X‐ray absorption spectroscopy with tuneable radiation is an important technique to investigate catalysts during operation, e. g., to study element‐specific changes in the structure (particles size, coordination) and chemical/electronic state during individual reaction steps or as a function of time on stream during reaction. Here, we will focus on XANES measurements at the L_III_ edges of both materials (Au and Ce), which offers insight into the chemical state of the Au/CeO_2_ catalyst via recording the electronic structure in the vicinity of the respective core levels. Due to the high absorbance of ceria in both energy regimes (Au L_III_ edge: 11919 eV, Ce L_III_ edge: 5723 eV), these measurements were carried out in the fluorescence mode (details on the measurements can be found in several publications[^28^, ^38^, ^46^, ^47^]).

#### 3.2.1 XANES at the Au L_III_ Edge

First, we will focus on the modifications at the Au L_III_ edge upon different pretreatments and during CO oxidation and water‐gas shift (WGS) reaction and discuss the influence of such processes on the chemical state of the Au NPs on the CeO_2_ support.

The pretreatment of the catalysts represents an important step in forming stable and uniform NPs from the as‐prepared Au/MO_x_ catalysts and is expected result in highly active and stable NPs. This step is however very decisive for the initial electronic state of the resulting catalysts.

**Figure 4:**
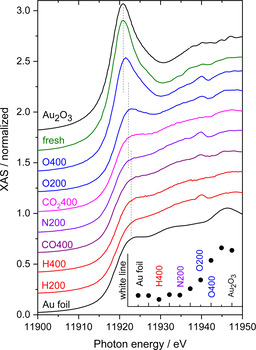
XANES spectra recorded at the Au L_III_ edge of Au/CeO_2_ catalysts before (fresh) and after different pretreatments. In black: data from metallic Au (Au foil) and Au_2_O_3_ references. Inset: corresponding white line intensities.

Figure 4 displays an overview of the chemical state of the Au NPs of the Au/CeO_2_ catalysts before (fresh) and after different catalyst pretreatments (abbreviations such as H400 mean: 10 % H_2_ in N_2_ at 1 bar and 400 °C), time periods are between 30 and 45 min. The respective measurements have usually been performed after the pretreatments at 80 °C/180 °C due to the high temperature during pretreatment and the close distance to the fluorescence detector with its sensitive beryllium window at the entrance (see more details in Refs [28,38,46,47]). Additionally, we included two XANES spectra (both in black) from metallic Au (Au‐foil) and Au_2_O_3_ powder as references for the evaluation of the Au spectra. The inset in the lower part of Figure 4 shows the magnitude of the white line for the different pretreatments and references.

**Scheme 1:**
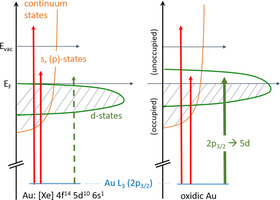
Schematic drawing of the electronic excitation from Au L_III_ edge into unoccupied states above the Fermi level (left part: metallic Au, right oxidic Au). The vertical green line in the right part represents transitions leading to pronounced “white lines” at the absorption edge. For metallic Au, this transition is not possible since all Au 5d states are occupied.

The experimental data recorded on the Au/CeO_2_ powder catalysts before the pretreatment (denoted as fresh catalyst) show a huge similarity with the data obtained from the Au_2_O_3_ reference. In both cases, the main feature is the pronounced white line directly at the absorption Au L_III_ edge exhibiting approximately the same shape and intensity. As indicated in the schematic drawing in Scheme 1, this feature arises due to excitations into the unoccupied Au 5d‐states of oxidic Au species[^63^, ^64^, ^65^] and consequently, the magnitude of the white line is proportional to the number of unoccupied states. The nearly identical intensity found for the fresh catalyst and Au_2_O_3_ was expected from the preparation methods (cf. experimental section), where HAuCl_4_ has been used as a precursor for the Au NPs.

A Linear Combination analysis (LCA) based on two references (Au‐foil and Au_2_O_3_) would be a typical approach to determine the degree of oxidation. It assumes, however, that the samples consist of a mixture of pure bulk‐like metallic Au and Au_2_O_3_ species. We applied this method to analyse the amount of Ce^3+^ species in the CeO_2_ support, where the remaining part of Ce is in the Ce^4+^ state (cf. following subsection). Following the work of Pantelouris,^[64]^ we used a fit for the Au L_III_ edge that includes a single symmetric peak for the white line and arctangent function for the transition into continuum states, for details, see ref. [38].

Based on this model the intensity of the Au L_III_ white line contribution of the different pretreatments is displayed in the inset in the lower part of Figure 4. After an oxidative (O400) pretreatment, the white line contribution decreases significantly in intensity compared to the fresh state, i. e., the Au NPs are present in a not completely oxidized state. This results fits well to Au 4 f XP spectra (Figure 2a) displaying about 70 % (either Au^1+^ or Au^3+^) oxidic Au species. The data after an O200 treatment show a much lower overall oxidation state compared to the O400 pretreatment indicating that the temperature has a significant influence on the final oxidation state. Pretreatments with gases that are weakly oxidative (CO_2_) were found to lead to a strong reduction of the white line compared to the oxidative treatments (O400 and O200). In case of a thermal treatment at 200 °C in an inert gas (N200), we observe a situation that is comparable to pretreatments with reducing gas species (CO and H_2_), which leads to the formation of the reduced (metallic) Au NPs. This indicates that the decomposition of oxidic gold species on the fresh catalyst in inert gases is mainly thermally induced. We cannot rule out the presence of Au^δ−^ in case of strongly reducing conditions at higher temperature (H400).^[64]^

**Figure 5:**
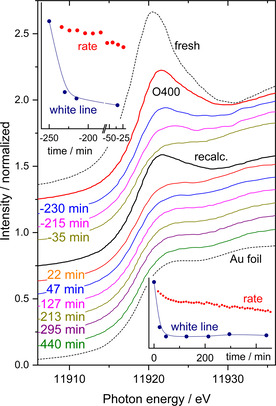
XANES spectra recorded at the Au L_III_ edge of Au/CeO_2_ catalysts during CO oxidation in 1 % CO, 1 % O_2_, balance N_2_ at 80 °C: Top part: after a first O400 pretreatment and (middle) after a second O400 pretreatments (recalcination). Spectra (in black, dotted) refer to metallic Au (Au foil, bottom) and the fresh catalyst (top). Insets: White line intensities (dark blue) and reaction rate (red) for the first O400 (top, left) and the second treatment (bottom, right).

Figure 5 displays XANES spectra recorded during CO oxidation and an additional recalcination process to recover the initial activity of the catalyst. The XANES spectra show pronounced white lines for the native Au/CeO_2_ catalyst and after the first O400 pretreatment (top of Figure 5) followed by a set of spectra with strongly decreasing white line intensity (curves in blue, magenta, and olive below the red O400 spectrum and inset in the top). A subsequent recalcination (second O400 treatment) leads again to an increase in the white line intensity, however, well below the level of the first O400 treatment. The spectra recorded after this process (between 45 and 450 min, cf. inset in the lower part of Figure 5) do not show any significant white line intensity, reflecting a complete reduction of oxidic Au species.

A more detailed analysis of the XANES data at the Au L_III_ edge displays a rather fast decay of the amount of oxidic species during time on stream, after 30 min, the peak area (and thus the amount of oxidic species) has dropped to less than 50 % of the initial amount. This loss in the oxidic character is not correlated to the much lower decrease in the reaction rate of such catalysts (cf. red data points in the insets in Figure 5), which is roughly 30 % of the catalyst initial activity within 15 h (note: here, we present only the beginning of the deactivation, for details, see ref. [46]). The deactivation was explained by a combination of a modest Au NPs growth, slow modifications in the support (oxidation of Ce^3+^ sites), and, to a larger extent, by accumulation of site blocking species.[^38^, ^46^, ^47^, ^54^]

When trying to re‐activate the catalysts in a further O400 treatment (recalcination), we find, as expected, an increase in the oxidic species, however, about 40 % lower than in the initial calcination process. Moreover, the loss of the oxidic character happens on a very fast time scale (again much faster than the decrease in the reaction rate, cf. inset in the lower part of Figure 5; red dots). The white line intensity decreases by approximately 50 % within 30 min and finally reaches a constant value (similar to the value of the metallic Au reference) after ∼1 h.

#### 3.2.2 XANES at Ce L_III_ Edge

To get insight into the effect of pretreatment and subsequent reaction gas mixtures on the catalyst support, which was not possible with ex‐situ XPS measurements (cf. Figure 3), we also applied operando XAS measurements at the Ce L_III_ edge to study modifications in the ceria support. Focusing first on the pretreatment effect we can also see a pronounced effect of the treatment gas on the resulting chemical properties of CeO_2_. While the oxidative pretreatment (O400) resulted in about perfect CeO_2_ structure (Ce exists mainly as Ce^4+^), with a negligible Ce^3+^ ions concentration, XANES spectra at the Ce L_III_ edge showed a notable reduction of CeO_2_ both after the reductive CO400 and the H400 pretreatments.^[54]^

**Figure 6:**
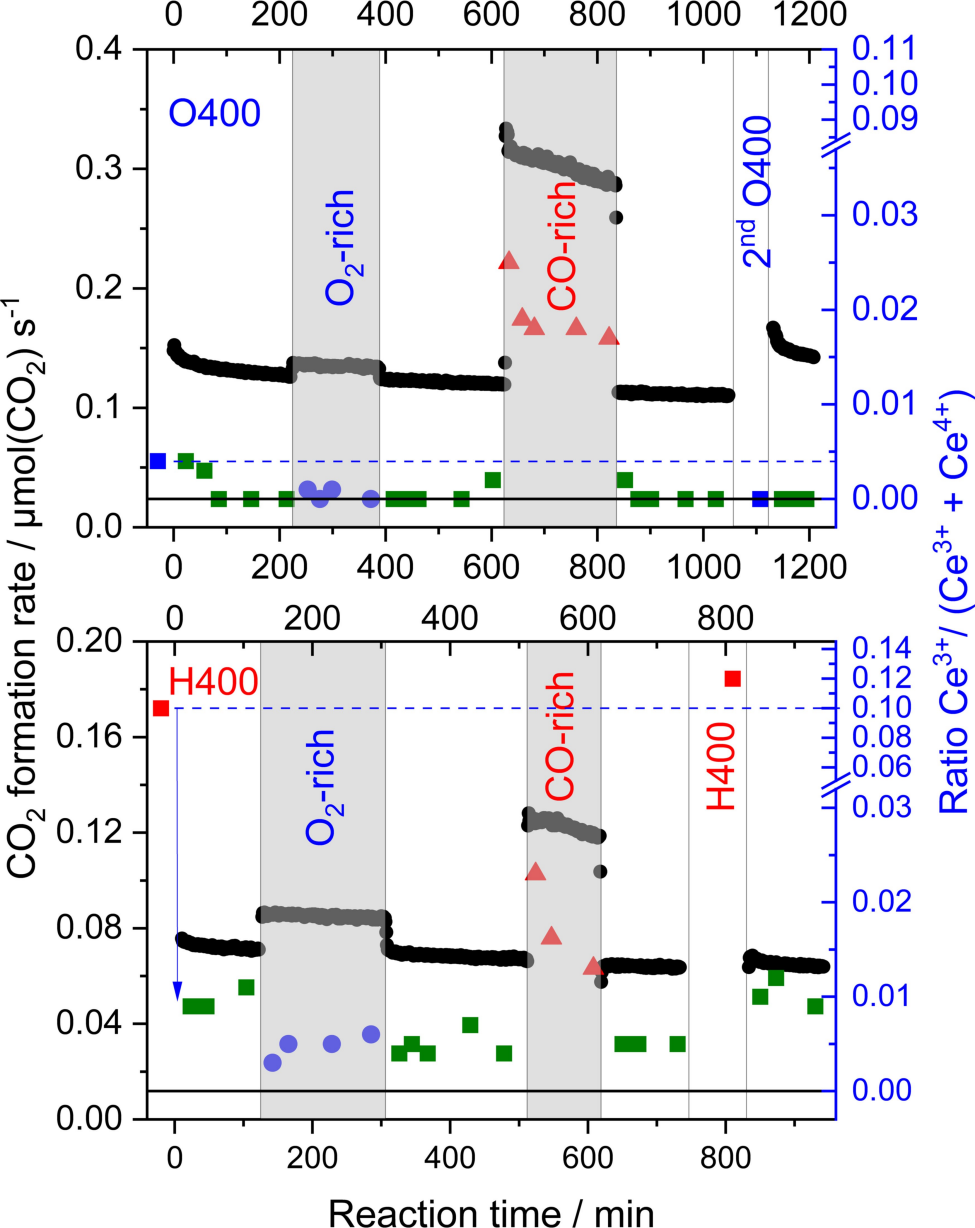
Formation rate during CO oxidation in diﬀerent reaction atmospheres (standard, O_2_‐rich, and CO‐rich) at 80 °C on a Au/CeO_2_ catalyst after oxidative (O400, top) and reductive (H400, bottom) pretreatment in 10 % O_2_ in N_2_ at 400 °C (black circles) and the relative Ce^3+^ content during time on stream as extracted from LCA analysis of operando XANES spectra during reaction (blue, green, and red symbols). The dashed blue line denotes the initial Ce^3+^ ratio (right scale). Representative error bars are shown for each sequence. Reprinted with permission from Ref. [54] © 2019, MDPI.

Based on a Linear Combination Analysis, we determined the formation of about 6 % and 10 % of the Ce^3+^ ions in the catalyst region accessible by XANES after reductive pretreatment. The fraction of Ce^3+^ and thus the O‐vacancies formed during a reaction such as CO oxidation is, however, strongly dependent on the composition of reaction gas mixture as shown in Figure 6 (right scale). For both pretreatments (O400 and H400) we observed a strong increase in Ce^3+^ species during CO rich gas atmospheres, whereas otherwise the fraction of this species stayed on a rather low level. Earlier results from *in situ* time‐resolved DRIFTS measurements also confirm the formation of Ce^3+^ defects during treatment,[^28^, ^38^] (for details, see section 5).

During CO oxidation in O_2_‐rich gas mixture at 80 °C, most of the Ce^3+^ species are rapidly re‐oxidized during the first few minutes of reaction, independent of the initial pretreatment (cf. Figure 6). For the O400 pretreatment the CO oxidation reaction leads to nearly a complete re‐oxidation of the pre‐existing Ce^3+^ species/O‐vacancies. For the reductively pretreated catalysts (in Figure 6, only H400 is shown), we can still quantify a small fraction of Ce^3+^ species/O‐vacancies (∼0.5 %), which is apparently resistant to re‐oxidation under present reaction conditions. This was tentatively explained by the presence of defects deeper in the support, which can be created during pretreatment at 400 °C because of the higher thermal mobility of bulk oxygen to the surface and its removal by reaction with hydrogen.^[56]^ These bulk defects are metastable during reaction at 80 °C due to the limited mobility of O‐vacancies under these conditions. In other words, if the catalyst is exposed to oxygen at 400 °C, we should expect a refilling of these bulk O‐vacancies. In CO‐rich gas mixtures, we observe a measurably higher fraction of Ce^3+^ compared to the O_2_‐rich gas mixture, which correlated to a higher steady state CO oxidation activity. Finally, it should be noted that the rate of decay of Ce^3+^ during reaction, after reductive treatment, is much faster than the rate of the catalyst deactivation. This implies that the Ce^3+^ concentration is not the only parameter deciding the catalytic activity of Au/CeO_2_ and again the concept of a combination of parameters contributing unequally to the overall activity is correct.

## 4 Au Particle Size Distribution During Pre‐treatments and Reaction

Determining the size and dispersion of Au NPs of supported catalysts is an indispensable requirement to understand their catalytic behavior. For Au/CeO_2_ catalysts this task is a real challenge due to the chemical nature of these catalysts. In most cases the limitations arise from the lack of sensitivity/resolution of the standard characterization techniques used in the assessment of the Au particle size such as standard X‐ray diffraction (XRD) or TEM measurements when the Au NPs are well below the cutoff limit of detection of these instruments. Recently, this problem was addressed by using high resolution aberration‐corrected scanning transmission electron microscopes (STEM), which could at least supply a more realistic view on the sub‐nanometer Au species.^[7]^ Additionally, the poor material contrast between CeO_2_ and Au complicates the analysis, especially for the high surface area CeO_2_ supports.[^27^, ^28^]

This aspect becomes more problematic when a detailed particle size or particle shape analysis is required for a detailed understanding of, e. g., deactivation processes. A possible misinterpretation of such microscopy or diffraction results is discussed in the following. First, small Au NPs present already in the fresh catalyst and/or after pretreatment may only grow during the reaction to a size below the detection limit of the standard (S)TEM (<1 nm) or XRD (<2 nm). In this case we will detect no change in the average size/size distribution of the Au NPs. The second and most complex situation is that particles grow just to the detection limit but remain below the initial average of Au NPs (e. g., average particle size is 1.5 nm and small particles grow to 1 nm). Consequently, the particle size distribution will shift downwards. In other words, the fraction of very small particles contributing to the particle size distribution will increase. The last situation would involve the growth of the sub‐nm sizes to values above the initial average size, where they can be detected by standard techniques (XRD/TEM): In this case, which reflects most studies in the literature about particles growth, we will observe an upshift in the average particle size.

Studies focusing on the low temperature water gas shift (LT‐WGS: CO+H_2_O⇌CO_2_+H_2_) showed partly contradictory trends in the Au particle size during reaction and their correlation with deactivation. Luengnaruemitchai et al. reported a modest increase of the Au nanoparticle size from 4 nm (obtained after an oxidative pretreatment) to 5.5 nm after reaction for about 3000 min in a water‐rich gas mixture (2 % CO, 20 % H_2_O and He).^[66]^ In contrast, Andreeva et al. found a completely different behavior, a decrease of the average Au particle size from 5.5 to 4.5 nm after reaction. The reaction conditions were rather similar in both studies, except for a reductive pretreatment in the latter case.^[67]^

Based on XRD/TEM results, Behm and coworkers showed, that the Au NP size distribution stayed almost constant (around 2 to 2.5 nm, error bar ±0.7 nm) in the LT‐WGS reaction under different reaction atmospheres in case of a H200 pretreatment. The deactivation was attributed to a buildup of thermally stable monodentate carbonate species from CO_2_ in feed gases.[^27^, ^40^] An increase of the Au NP size was also excluded for an oxidative pretreatment (O400) followed by subsequent LT‐WGS reaction at 180 °C.^[27]^ Later on, the Behm group employed operando EXAFS studies on differently pretreated Au/CeO_2_ catalysts to gain a more accurate insight into this controversial aspect. Different oxidative and reductive pretreatments can significantly influence the electronic state of the Au species (Au^δ−^, Au^0^, Au^δ+^). Exposure to the reaction atmosphere leads to the rapid formation of extremely small, sub‐nm sized Au^0^ NPs,^[28]^ which are the dominant Au species during LT‐WGS and responsible for the high catalytic activity. The deactivation remains, however, mainly related to the buildup of carbonate species.^[27]^

Obviously, the behavior of Au/CeO_2_ catalysts during LT‐WGS is not much different for the CO oxidation. The catalysts first show a very fast activation phase followed by a subsequent continuous deactivation with time on stream. To decide if there is a common factor responsible for deactivation in both reactions, we also combined results from operando EXAFS measurements with high resolution electron microscopy (HAADF‐STEM measurements) during CO oxidation, in addition to XRD results (HAADF: high angle annular dark‐field imaging). The results from these three techniques may, on the first glance, show different pictures. Considering, however, the limitations and sensitivities of the methods, a careful merging of all results enables the more detailed view on the particle size distribution.

**Figure 7:**
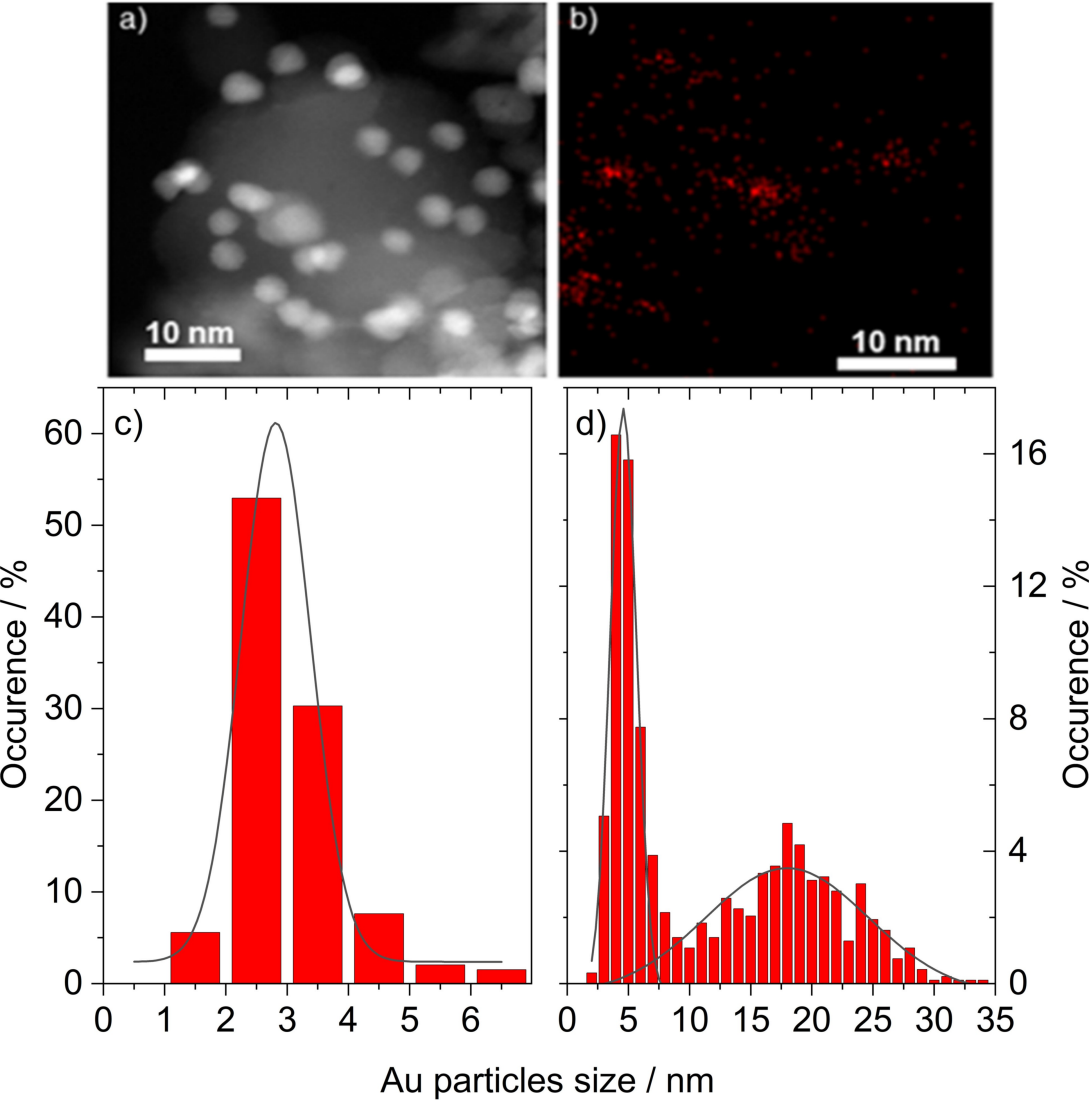
a) STEM micrograph after O400 treatment and b) corresponding EDS map. c) Particle size distribution based on STEM/TEM analysis of Au/CeO_2_ catalyst after O400 pretreatment and (d) after additional CO oxidation. c) and d): Reprinted with permission from Ref. [38] © 2016, Elsevier Inc.

As an example, for a 4.5 wt.% Au/CeO_2_ catalyst, XRD results indicated that the exposure of the Au/CeO_2_ catalyst to an oxidative pretreatment (O400) and subsequent CO oxidation (1 % CO, 1 % O_2_, balance N_2_) at 80 °C for 1000 min on‐stream results in a modest increase of the average Au particle diameter from 2.2±0.5 nm (O400) to 3.4±0.8 nm (CO oxidation).^38^ A reductive H400 pretreatment resulted in slightly smaller Au NPs with a diameter of 1.8±0.5 nm, which remained unchanged during reaction. Upon performing the pretreatment in CO (CO400), the particle size remained mainly unchanged (2.6±0.5 nm before, 3.0±0.5 nm after CO oxidation) after exposure to the CO oxidation. On the contrary, a HAADF‐STEM investigation of Au/CeO_2_ samples after an O400 treatment showed that the particle size distribution is in the range from 1 to 8 nm (see Figure 7a,c), slight differences in the particle size distribution can be observed depending on the Au/CeO_2_ batch (even when using the same method of preparation and under strict control of preparation parameters).[^38^, ^41^] The average size is about 3.4±0.8 nm and thus slightly larger than the average size observed by XRD after the same pretreatment (O400). With a more detailed analysis based on STEM‐EDS mapping, we could clearly see that a significant fraction of Au NPs exists also in the sub‐nanometer size range as small clusters and single atoms together with nanoparticles in the size range of 2–5 nm. After CO oxidation the STEM results showed a bimodal distribution of Au NPs with maxima at 4.5±2.1 nm and a broad maximum around 18 nm.^[38]^ This observation differs from the Au particle size extracted from XRD data (based on the Scherrer equation) which revealed already a limited growth of the mean Au NPs during reaction. Obviously, XRD is also not sensitive to large particles in the presence of many smaller ones.

**Figure 8:**
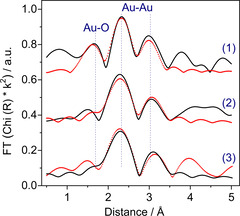
Magnitude of the k^2^‐weighted Fourier transform converted EXAFS data (solid black lines) and corresponding fits (red dots) for a 4.6 wt.% Au/CeO_2_ catalyst during CO oxidation reaction in (1 % CO, 2 % O_2_ & 98 % N_2_) at 80°after O400 pretreatment. Note: these data are not phase‐corrected and thus, the distances are smaller than realistic Au−Au and Au−O distances. The spectra listed follow the order: (1): in N_2_ directly after calcination at 80 °C; (2): 65 min CO oxidation; (3):175 min CO oxidation.

Additionally, we performed EXAFS measurements directly after the catalyst activation and during the subsequent CO oxidation.[^38^, ^46^] After the O400 treatment one can observe partially oxidized Au nanoparticle with two contributions characteristic for Au−O and Au−Au back scattering shells. The respective Au−O contribution should appear at a distance of about 2.0 Å. It is, however, usual to present these EXAFS data without phase‐correction which generally leads to smaller distances as, e. g., visible for the Au−O distance in Figure 8.

This contribution was found to decrease significantly during the first 60 min of reaction and almost no measurable contribution could be observed at a period longer than 65 min during CO oxidation on stream (see Figure 8),[^68^, ^69^] where at the same time an increase of the magnitude of the first neighbor Au−Au coordination number was observed, from 6.3 (after pretreatment) to about 8.0 (after 175 min during reaction on stream).^[38]^

This rather small change in the Au particle size obtained by EXAFS data analysis cannot explain the presence of the huge Au NPs found in the STEM images after reaction, neither in CO oxidation nor in the WGS reaction. EXAFS is highly sensitive to very small particles, typically smaller than 2–3 nm, i. e., nanoparticles close or below the detection limit of XRD and conventional TEM. A plausible explanation would be that a very small fraction of the highly dispersed Au species (<5 % of all particles) present on the pretreated catalyst has grown during reaction forming these huge Au NPs, while the majority of the dispersed metal only showed a moderate growth in the size range of a few nm. Here, it should be highlighted that EXAFS results showed much smaller coordination numbers after reductive pretreatments (3.4±0.3 and 3.9±0.4 for H400 and CO400), which would correspond to a dispersion above 90 %. This scenario means that not only Au NPs or sub‐nm clusters would exist on the surface of CeO_2_, but also atomic Au species, which were assumed as active species for the CO oxidation by Zhang and coworkers.^[70]^ Using operando FTIR and DFT calculations, Schilling et. al. showed recently that isolated Au single sites, identified as O_lattice_‐Au^1+^−CO species, are monitored during reaction and can be assumed as active Au species during CO oxidation.^[71]^ Particle growth during reaction was also reported as a possible reason for the deactivation of Au/CeO_2_ catalysts,^[35]^ similarly to Au/TiO_2_.^[72]^

Based on all these studies we assume that Au NPs of a rather broad sizes distribution contribute to the activity, however, to different extents. Thus particle growth/sintering with time on stream will affect the activity, but is not solely responsible for the deactivation. This does not mean that, for instance, single atoms are more active than small clusters or vice versa. There might be an optimum size/and oxidation state responsible for the initial high activity which changes with time on stream and interacts with other changes in the support. Parameters that might be responsible for the modifications will be discussed below.

## 5 Insights From Raman and FTIR Spectroscopy

The defect sites in CeO_2_ supports (i. e., oxygen vacancies or Ce^3+^ species) play a decisive role in the activity of Au/CeO_2_ catalysts for different reactions. This aspect had been investigated in several groups using operando spectroscopy techniques, especially Raman spectroscopy, which gave valuable insights into the nature and role of these species.[^73^, ^74^, ^75^, ^76^, ^77^] To date, it is accepted that the activation of O_2_ depends on the presence (formation) of O‐defects (O‐vacancies).[^73^, ^74^, ^75^] The formation of these point defects in CeO_2_ is highly dependent on the catalyst pretreatment.[^38^, ^54^, ^77^] Different from the XANES measurements, Raman spectroscopy is more sensitive to changes in the chemical composition of CeO_2_ and its chemical environment, in addition to providing more quantitative information.

Recently, Hess and coworkers intensively used *in‐situ*/operando Raman spectroscopy for the characterization of CeO_2_ based materials during CO oxidation.[^76^, ^77^, ^78^] They demonstrated that the extent of reduction of the ceria support has a significant influence on the activity of Au/CeO_2_ catalysts.^[77]^ They observed about two times higher activities in the CO oxidation on the pre‐reduced catalyst (in 2 % CO/Ar) compared to catalysts being pre‐oxidized (in 25 % O_2_/Ar). The subsurface reduction of CeO_2_ (i. e., formation of bulk O‐defects) during reaction was monitored by operando Raman and UV‐Vis spectroscopy. These authors observed a clear red‐shift of the F_2g_ band, which is indicative for the increase of the concentration of O‐vacancies.^[77]^

**Figure 9:**
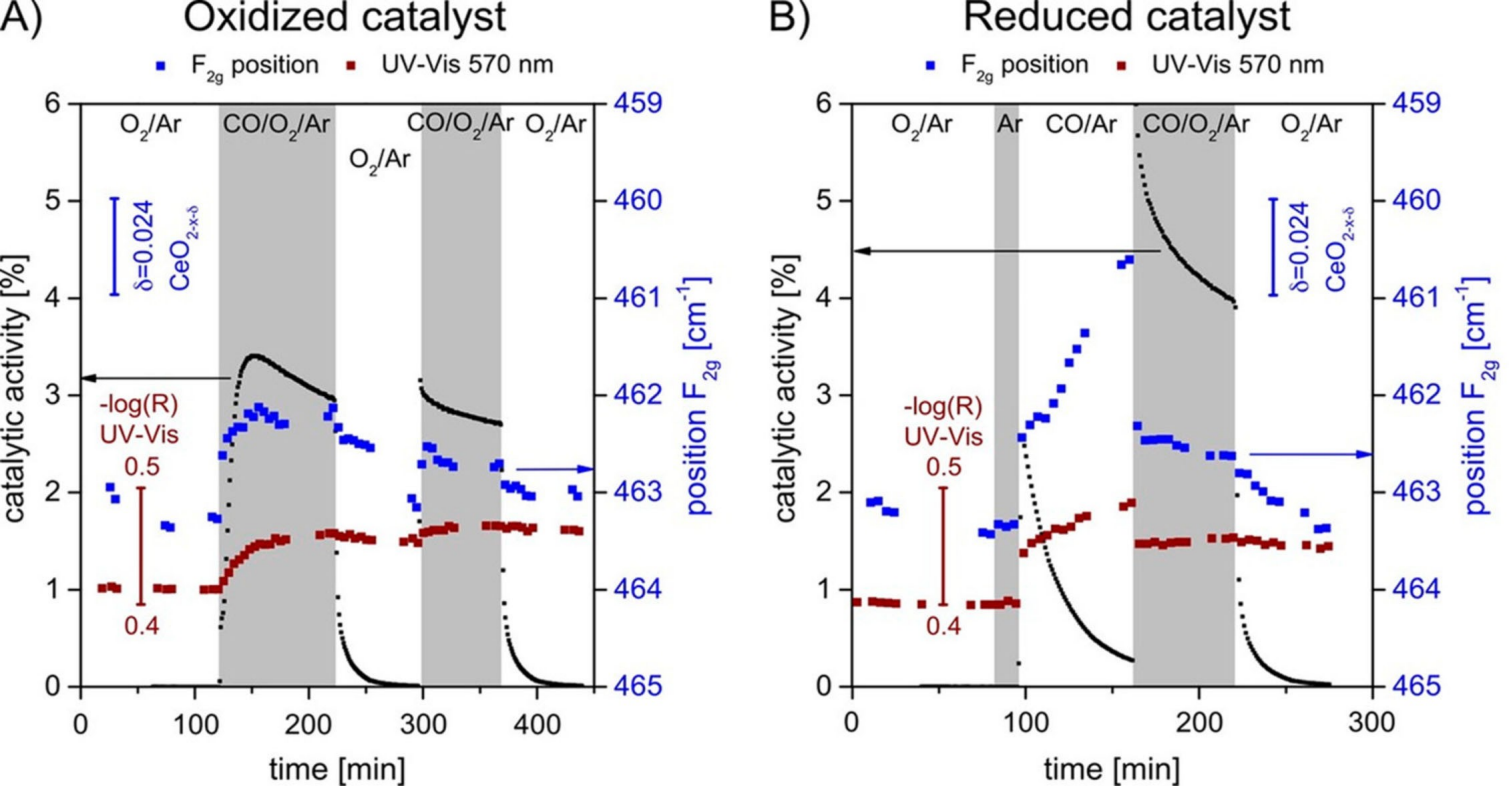
Time‐dependent Raman and UV‐vis operando spectroscopic information for the ceria subsurface reduction state of 0.5 wt.% Au/CeO_2_. A): During two consecutive exposures to reaction conditions after oxidative pretreatment. B) During reaction after reductive pretreatment. Black squares indicate the catalytic activity of the catalyst, blue squares the position of the F_2g_ Raman mode, and brown squares the UV‐Vis reflectivity at 570 nm. The scales are identical for both graphs. Reprinted with permission from Ref. [77] © 2018, American Chemical Society.

For the oxidatively treated Au/CeO_2_, a red‐shift of the F_2g_ band (463.6 cm^−1^) by 1.5 cm^−1^ was observed upon exposure to CO oxidation reaction gas (Figure 9A, note the shift of the blue dots representing the position of the F_2g_ mode). This typical F_2g_ red‐shift correlates to a change of the stoichiometry of ceria (from CeO_1.988−x_ after the oxidative treatment to CeO_1.952−x_) during the CO oxidation. This refers to a reaction induced reduction of the catalyst support upon interaction with CO. After a reductive treatment, a red‐shift of the F_2g_ band by about 3 cm^−1^ was observed (during exposure to CO/Ar), which is equivalent to a stoichiometry of CeO_1.916−x_. When switching to the reaction gas, the ceria reaches the same equilibrium composition as observed after an oxidative step (CeO_1.952−x_) (Figure 9B, blue dots in the grey area). According to these findings, the higher catalytic activity after a reducing pretreatment is largely attributed to the subsurface reduction of the ceria support. This result hints at the formation of subsurface O‐vacancies, which agrees well with the conclusions obtained from the evaluation of Ce L_III_ spectra under comparable conditions (see section 3).

Furthermore, these results agree well with recent findings from the Behm group on Au/ZnO catalysts when exposed to CO_2_/H_2_ mixtures under strongly reducing conditions at temperatures ≥240 °C.[^79^, ^80^] Independent of the initial concentration of O‐defects, the catalyst reaches a similar steady state activity, which is closely related to the reaction gas atmosphere (e. g., more Ce^3+^ can be observed in CO‐rich than in O_2_‐rich reaction gas, cf. Figure 6).^[54]^ In general, this means that the concentration of Ce^3+^ ions and thus of O‐vacancies in the surface region equilibrates rather quickly when being exposed to the reaction gas.

*In situ* FTIR measurements supplied additional evidence on the formation of Ce^3+^ species. For Au/CeO_2_ catalysts after H400 pretreatment, we observed a distinct band in the C−O stretch region at 2125 cm^−1^, which may be attributed to the formation of Au−H species.[^38^, ^82^] This band was found to rapidly disappear upon exposure to O_2_ at room temperature. However, we also observed a signal at this frequency on the pure CeO_2_ support.^[38]^ A similar disappearing signal was also found after reduction in H_2_ (2.5 wt.% Au/CeO_2_ catalyst) upon exposure to a dilute water gas mixture (1 % CO+2 % H_2_O vapor, balance N_2_). This feature was assigned to a “forbidden” ^2^F_5/2_ to ^2^F_7/2_ transition being characteristic for Ce^3+^ ions,^81^ which are formed by reduction of the CeO_2_ support during exposure to H_2_/N_2_ at temperatures between 200 and 400 °C. The evolution of this band during CO_2_ reduction on Au/CeO_2_ catalysts could be monitored by high pressure *in situ* DRIFTS and indicated a possible overreduction of the support under those conditions.^41^

**Figure 10:**
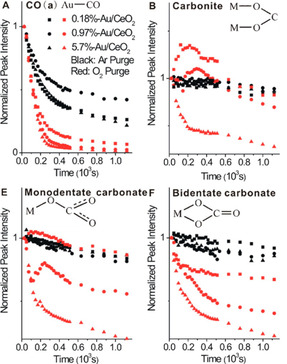
Variations of the normalized vibrational peak intensities of major surface species formed on various Au/CeO_2_ catalysts upon saturating CO adsorption at room temperature as a function of time during the initial purging processes in Ar and O_2_. Reprinted with permission from Ref. [49] © 2015, American Chemical Society.

A further interesting aspect observed by operando FTIR measurements on Au/CeO_2_ catalysts is the dependence of the CO oxidation reaction pathway on the Au particle size.^[49]^ Chen et al. employed time‐resolved operando DRIFTS measurements to monitor the time evolution of various carbonaceous surface species (e. g. carbonates and formates) during CO oxidation at room temperature on different Au/CeO_2_ catalysts where the average Au NP size was varied in the range from 1.7±0.6 nm to 3.7±0.9 nm (cf. Figure 10). The evolution of these surface species on Au/CeO_2_ catalysts was monitored after an initial CO saturation at RT during subsequent purging processes in Ar and O_2_, respectively. The results obtained from CO_ad_ species and different carbon containing intermediates (surface carbonates and bicarbonates) indicated that the intrinsic oxidation activity of CO_ad_ species does not significantly depend on the Au particle size (Figure 10a). On the contrary, the activity of the oxygen‐assisted decomposition of adsorbed carbonate and bicarbonate species is strongly dependent on the Au particle size and favored over larger Au NPs (Figure 10b–c). It seems that larger Au NPs (between 2.6 and 3.7 nm) are more capable of activating surface lattice oxygen on CeO_2_ to participate in the CO oxidation via surface intermediates such as carbonates, bicarbonates, and formates. This phenomenon may be connected to the earlier findings by Valden and Goodman, where the highest CO oxidation activity was observed for Au NPs with a size of about 3.0 nm.^[24]^

A possible explanation could be the difference in the O_2_‐assisted decomposition of (bi‐)carbonates caused by different concentrations of the available surface oxygen species, which, in turn, depends on the Au particle size for these catalysts. This parameter can affect the activation of molecular oxygen and its diffusion to the Au‐ceria interface. Nevertheless, this aspect needs to be investigated in more detail to assess the impact of the Au particle size on the formation/diffusion of O‐defects on ceria and thus its influence on the CO oxidation activity.

## 6 TAP‐Reactor Studies on Au/CeO_2_

Finally, we address the oxygen storage capacity (OSC) of Au/CeO_2_ using the technique of Temporal Analysis of Products (TAP), which is able to determine the amount of oxygen stored on and reversibly removed from the catalyst. For these measurements we normally employ oxidizing (CO_2_ or O_2_) or reducing (CO or H_2_) gas pulses of 8.0×10^15^–1.0×10^16^ molecules per pulse, which is an infinitesimal amount of gas hitting the catalyst surface. Gas molecules from the pulses passing through the catalyst bed, being desorbed from the catalyst or produced in the catalytic reaction, are detected by a quadrupole mass spectrometer. Compared to microkinetic and spectroscopic measurements, the results obtained from TAP reactor titration have the advantage of being conducted under non‐destructive conditions of the catalyst (the catalyst surface is largely retained during measurements).

In the activity measurements, where CO and O_2_ were pulsed simultaneously, one can evaluate the conversion of both reactants for each single pulse. Hereby, we are able to obtain insights into the activation behavior of the catalyst in the beginning of the reaction. This information is rather difficult to decipher from kinetic measurements due to the fast changes occurring on the surface of the catalyst upon exposure to reaction gas mixtures, e. g., the substantial loss of O‐vacancies in CeO_2_ during CO oxidation once exposed to CO/O_2_ gas mixture.

This can be seen and quantified from TAP measurements with much higher time resolution, which was not possible from other spectroscopic techniques. In particular, those measurements have been very informative about the dynamics of formation of O‐vacancies in the ceria lattice and replenishment by gas phase oxygen. This phenomenon has been addressed by multi‐pulse experiments in which lattice oxygen from the Au/CeO_2_ catalyst was reversibly removed by CO pulse sequences and replenished in subsequent O_2_ pulses.

**Figure 11:**
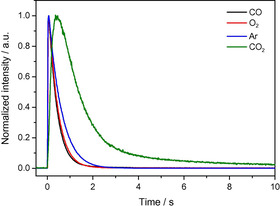
QMS single pulse response of the height‐normalized signals of CO (m/z=28), O_2_ (m/z=32), Ar (m/z=40) and CO_2_ (m/z=44) on the pre‐oxidized (O400 treatment: 1 h in 10 % O_2_/N_2_ at 400 °C) Au/CeO_2_ catalyst during simultaneous pulses with CO/Ar and O_2_/Ar at 180 °C. For better comparison the QMS mass signals were normalized to the same height Reprinted with permission from Ref. [83] © 2013, Elsevier Inc.

Figure 11 illustrates the QMS detected single pulse response of CO, O_2_, Ar, and CO_2_ in a simultaneous pulse of O_2_/Ar and CO/Ar on the completely pre‐oxidized Au/CeO_2_ catalyst at 180 °C. The signals presented here were detected after reaching a constant value of CO/Ar and O_2_/Ar pulse signals at the end of the measurement.^[83]^ The typical pulse shape of a steep increase followed by an exponential decay of the signal was detected for all species: The observed broadening of the CO and O_2_ pulses is almost identical to that of Ar resulting in a pulse width of about 2 s. Based on the inert behavior of Ar we conclude that the broadening of CO and O_2_ mainly results from transport processes of the unreacted gas through the catalyst bed. The CO_2_ pulse, in contrast, showed a larger broadening of approx. 10 s. Additionally, the CO_2_ peak width was found to strongly depend on the reaction temperature and the number of CO_2_ molecules per pulse. Thus, strongly adsorbed CO_2_ on the catalyst bed or CO_2_ containing surface carbonate species may be formed and decomposed during the simultaneous CO and O_2_ pulses.^[83]^

**Figure 12:**
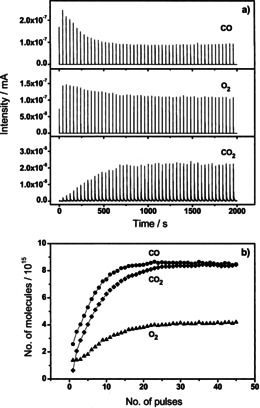
a) Sequence of simultaneous CO and O_2_ pulses dosed on a Au/CeO_2_ catalyst pretreated by calcination in 10 % O_2_/N_2_ at 400 °C (30 min); (b) CO uptake (•), oxygen uptake (▴) and CO_2_ formation (⧫) during these pulses. Reprinted with permission from Ref. [36] © 2007, Elsevier Inc.

As mentioned before, we followed the activation process in a complete sequence of simultaneous CO/Ar and O_2_/Ar pulses directly after the O400 pretreatment. The mass spectrometric signals for CO, O_2_ and CO_2_ in a sequence of 50 simultaneous gas pulses (Δt=40 sec) of O_2_/Ar and CO/Ar at 120 °C are presented in Figure 12a.^[36]^ After integration of the signal area, the amounts of O_2_ and CO consumed, and CO_2_ released were quantitatively determined for each pulse. For a sequence of 50 simultaneous pulses (Δt=40 sec) of O_2_/Ar(1 : 1) and CO/Ar(1 : 1) at 120 °C, the results are shown in Figure 12b.^[36]^ After an activation phase of ∼30 consecutive pulses a steady state was reached, in which we observed a stoichiometric O_2_ and CO consumption quantitatively related to CO_2_ formation. During this phase we noticed that the CO consumption was higher than that of O_2_ and the CO_2_ release.

These observations indicate that part of the formed CO_2_ remains stable on the catalyst surface. Subsequent TPD measurements indicated that the CO_2_ resulting from the reaction of CO with lattice oxygen (or the co‐fed O_2_) contribute to the formation of surface carbonate. The calculated deficit of CO_2_ desorption in the initial phase was 1.8×10^19^ molecules CO_2_ g_cat_^−1^ or 0.06 ML surface carbonate on the catalyst. On the other hand, given the initial CO consumption exceeding the O_2_ consumption, we conclude that CO not only reacted with O_2_ deposited on the catalyst from the gas phase, but also with surface lattice oxygen from the catalyst support. The amount of CO consumed by reaction with surface oxygen was calculated to be 2.3×10^19^ molecules CO g_cat_^−1^, which is equivalent to 0.07 ML surface oxygen. From this behavior we conclude that the pre‐oxidized surface of the catalyst was slightly reduced during the activation phase. Here we want to note that the observed initial reduction of the catalyst during the activation period can be correlated with:

- the fast reduction of the oxidic Au species (AuO_x_) which according to operando XANES/EXAFS measurements occurs in <2 min on‐stream under normal flow conditions (cf. section 4)[^28^, ^38^, ^47^] and
- the fast (2 min) partial reduction of the fully oxidized support as discerned by measurements at the Ce L_III_ edge during CO oxidation,^[54]^ and operando Raman spectroscopy measurements (discussed in detail in section 5).[^76^, ^77^, ^84^]

With a higher number of pulses in this sequence the ratio of CO and O_2_ consumption approached the stoichiometric conversion, which is equivalent to steady state condition.^[36]^ This observation also fits well with spectroscopic data, which showed no measurable changes of electronic or structural properties under steady state conditions.

**Figure 13:**
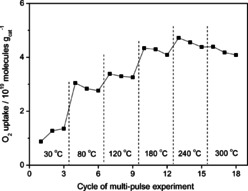
Effect of reaction temperature on O_2_ uptake during three alternating sequences of 100 O_2_/Ar pulses (Δt=5 s) after 1000 pulses of CO/Ar (Δt=1 s) on a Au/CeO_2_ catalyst. Reprinted with permission from Ref. [85] © 2015, The Royal Society of Chemistry.

In the alternating multi‐pulse experiments we have additionally investigated the ability to reversibly remove surface oxygen from the Au/CeO_2_ catalyst by CO and subsequently to replenish the oxygen vacancies with gas phase oxygen (see Figure 13). The oxygen storage capacity depending on the reaction temperature was quantitatively evaluated by determining the O_2_ consumption in a sequence of O_2_/Ar (1 : 1) pulses (from the relative pulse areas of O_2_ to Ar) compared to the situation in the end of a pulse sequence, considering under these conditions no further conversion taking place (for details, see Ref. [36,83,85]).

The O_2_ uptake in three consecutive titration cycles at temperatures between 30 °C and 300 °C, directly after a preceding reduction step using a sequence of 1000 CO/Ar pulses (Δt=1 s) are plotted in Figure 13.^[85]^ Between 30 °C and 180 °C the amount of O_2_ consumed as well as the amount of CO_2_ released increased strongly with increasing temperature, but became less pronounced between 180 °C and 240 °C. This observation indicates the ability of surface oxygen species to move to the Au‐ceria perimeter sites, which is not accessible from spectroscopic measurements. At 300 °C the O_2_ loss was slightly lower than at 240 °C; the increased oxygen diffusion from the bulk to the surface at higher temperatures may cause a partial re‐oxidation of the surface already during CO pulses, which reduces the amount of O_2_ necessary to completely oxidize the surface in the subsequent O_2_ pulse sequence.

From 80 °C upwards the titration experiments showed that the oxygen consumption at each reaction temperature decreased with the number of titration cycles, which is more pronounced between the first and second run. A possible explanation for this behavior is the accumulation of stable adsorbed carbonate species formed during CO pulses, which are already formed during simultaneous O_2_ and CO pulses. The formation of stable carbonate species on the catalyst surface leads to the blocking of active sites for oxygen removal by CO.^[85]^

In a comparable series of multi‐pulse experiments, we pulsed H_2_/Ar instead of CO/Ar to remove active oxygen from the catalyst (data not shown) at the same reaction temperatures as shown in Figure 13. We observed that 1000 pulses of CO are able to remove a larger amount of active oxygen from the catalyst than 1000 pulses H_2_. At 30 °C and 80 °C, H_2_ was not active in removing surface oxygen from the catalyst, above 80 °C the difference in the O_2_ removal by CO and H_2_ was highest at lower temperatures and decreased with higher reaction temperature. This underlines the higher efficiency of CO in reducing an oxidized Au/CeO_2_ catalyst compared to H_2_.^[85]^

The findings reported by Behm and co‐workers using TAP reactor surface titration measurements interestingly agree well with DFT calculations on the CO oxidation mechanism at the interface of CeO_2_ supported Au nanocrystals. Kim and Henkelman found that the energetically favored reaction mechanism is a Mars‐van Krevelen type. In this scenario an adsorbed CO molecule on the Au nanocrystal is directly oxidized by an oxygen atom protruding from the CeO_2_‐step sites at or close to the Au‐Ceria perimeter sites. The Au NPs supplies the CO molecule, whereas the oxygen atom is provided by the CeO_2_ support; as a consequence, both reactants do not compete for the same binding site.

Thus, a large portion of the CO oxidation activity on Au/CeO_2_ can be attributed to reaction at the interface of the CeO_2_ step and the Au NP.^[86]^ They also indicated that this process should be assisted by dynamic formation/presence of O‐vacancies in the CeO_x_ structure which act as anchor sites for the activation of O_2_ and the resupply of atomic species to the CO_ad_ species at the interface. These calculations^[87]^ agree quite well with spectroscopic results discussed above.

Using a different metal in combination with CeO_2_ (2.0 wt.% Pt/CeO_2_ catalyst) Shektman et al. investigated the CO oxidation using TAP reactor multi‐pulse experiments. At 300 °C, they determined the CO consumption in a pulse sequence after preceding oxidative pretreatment (20 % O_2_/N_2_, 1 h, 300 °C) to be 7.7×10^20^ molecules g_cat_^−1^,^[88]^ which is one order of magnitude higher than the steady state CO consumption on Au/CeO_2_.^[85]^ However, for Pt/CeO_2_ catalyst, the temperature dependence of the active oxygen removal during exposure of the pre‐oxidized catalyst to a CO pulse sequence resembles that one of Au/CeO_2_. The amount of oxygen removed by CO from the catalyst increased with higher temperature for both, CeO_2_ supported Pt and Au NPs. On top of that, for the CO oxidation on Pt/CeO_2_ catalyst, two different types of active sites were observed with a different temperature dependence of their activity. The more active sites with a lower dependence of the amount of oxygen stored and removed from the catalyst on the reaction temperature were attributed to the interface of Pt NPs and the CeO_2_ support and sites in close proximity. The other type of active sites for CO oxidation on Pt/CeO_2_ with a stronger dependence of the activity on the reaction temperature were supposed to be on the support, where the energy barrier of the reduction is higher compared to the Pt/CeO_2_ interface.^[88]^ Comparing these results with those for Au/CeO_2_, we conclude that the decisive active site for CO oxidation on Au/CeO_2_ may be the support‐NP interface, which is different from Pt/CeO_2_ where the support is strongly modified and involved in the reaction path.

## 7 Conclusions

In this review we showed results from XPS, different operando spectroscopic (XANES, EXAFS, Raman, FTIR), HR‐STEM and TAP reactor studies giving insight into catalytic performance of highly active Au/CeO_2_ catalysts. These results can be summarized as follows:

- Independent of the catalyst pretreatment, the exposure of Au/CeO_2_ catalyst to different reactants results in a modest increase of Au particle size, which includes the formation of small, but measurable fraction of huge particles (20–100 nm), sub‐nanometer Au NPs, as well as isolated single atoms.
- Different Au species (single atoms, sub‐nm clusters, NPs) are assumed to contribute with different weights to the accumulative reaction rates, while the debate about the (most) active Au species is still controversial and unsolved.
- Under CO oxidation/LT‐WGS reaction conditions, the dominant oxidation state of gold is zero (metallic Au). Higher oxidation states such as Au^1+^, Au^3+^ can be generated and observed after oxidative treatment, however, they largely vanish during reaction (CO oxidation, WGS) or reach very limited concentration stabilized in the sub‐surface region (e. g., Au^3+^ observed after O400 treatments).
- Ce^3+^ species (O‐vacancy defects) in near surface regions of the support surface can be generated by treatment at high temperatures and their concentration is higher in reductive gas mixtures. The formation of sub‐surface/bulk Ce^3+^ defects can be achieved in CeO_2_ and similar oxides upon reductive treatments at elevated temperatures and their concentration depends on the reducing power and temperature of treatment.
- Ce^3+^ sites could be observed both using Raman and XANES measurements during CO oxidation and WGS reactions where their concentration relies significantly on the ratio of CO/O_2_ or CO/H_2_O gas mixture. The concentration of Ce^3+^ species is related to the catalytic activity.
- Finally, the main reason for the deactivation of these catalysts can be correlated with a combination of effects (listed as: Au NP growth, overreduction of the catalyst by excess of Ce^3+^ sites or poisoning by surface carbon‐related species) whose relative effect depends largely on the reaction gas mixture.

In spite of the presence of a number of open questions in the catalytic performance of Au/CeO_2_ catalysts we would like to underline the important contribution of Prof. Jürgen Behm to the fundamental understanding of activity and deactivation processes of Au/MOx catalysts in prototype reactions in catalysis.

## Conflict of interest

The authors declare no conflict of interest.

## Biographical Information

*Ali Abdel‐Mageed studied chemistry and obtained his M.Sc. at Cairo University, Egypt, in 2009. In 2016, he got his PhD at Ulm University. He continued his work on heterogeneous catalysis as a group leader since 2017. His research focuses on studying heterogeneous catalysts in the storage and conversion of renewable energies employing micro‐kinetics and operando spectroscopy*.

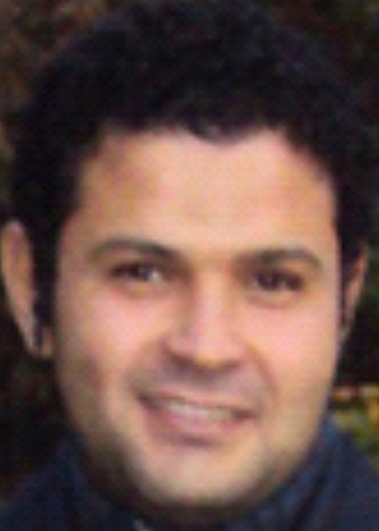

## Biographical Information

*Shilong Chen received his PhD from Univ. of Science and Technology of China (USTC) in 2016. He then worked at Ulm University as a postdoctoral researcher. Since Mar. 2021, he is a scientific staff member at Christian‐Albrechts‐Universität Kiel. His research interests focus on the selective conversion of CO_2_ to synthesized fuel*/*chemical and fundamental understanding of catalytic processes by operando spectroscopy*.

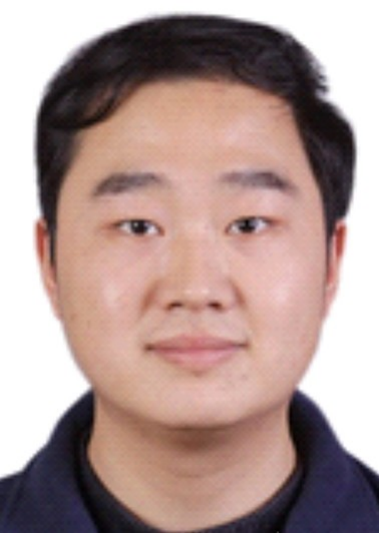

## Biographical Information

*Corinna Fauth received her M.Sc. in Chemistry and Management from Ulm University in 2019. Currently she is a PhD student at the Institute of Surface Chemistry and Catalysis. Her research mainly focuses on the dynamics of reversible oxygen storage and oxygen activation on different heterogeneous catalysts*.

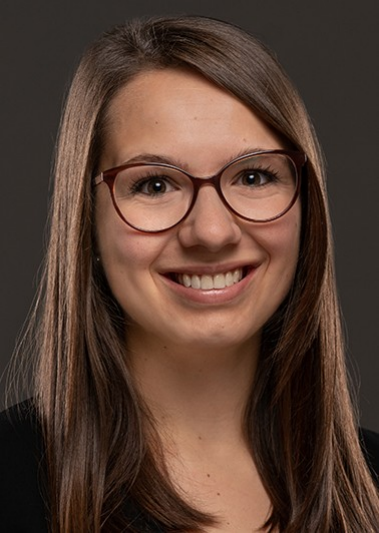

## Biographical Information

*Thomas Häring is a certified mechanical engineering technician. 1987–1991: Institute of Crystallography and Mineralogy (LMU München). 1991–1992: technician at Institute Laue‐Langevin, Grenoble (France). Since 1992: Technician at the Institute of Surface Chemistry and Catalysis (Ulm University). He developed the home made flow cell for operando spectroscopy using synchrotron radiation and participated in beam times*.

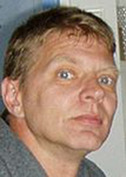

## Biographical Information

*Joachim Bansmann studied physics and obtained his PhD. at Univ. of Bielefeld (1993). He then moved to Rostock where he finished his habilitation in physics in 1999. Since 2005 he is group leader in the Institute of Surface Chemistry and Catalysis at Ulm University. His field of research is related to surface science (e. g. model catalysis), nanoparticles on surfaces, and applications of synchrotron‐based (operando) spectroscopy methods*.